# The Pseudo-Circular Genomes of Flaviviruses: Structures, Mechanisms, and Functions of Circularization

**DOI:** 10.3390/cells10030642

**Published:** 2021-03-13

**Authors:** Louis De Falco, Nelly M. Silva, Nuno C. Santos, Roland G. Huber, Ivo C. Martins

**Affiliations:** 1 Bioinformatics Institute (BII), Agency for Science, Technology and Research (A*STAR), 30 Biopolis Street, Matrix #07-01, Singapore 138671, Singapore; louisd@bii.a-star.edu.sg; 2 Instituto de Medicina Molecular, Faculdade de Medicina, Universidade de Lisboa, 1649-028 Lisbon, Portugal; nellysilva@medicina.ulisboa.pt (N.M.S.); nsantos@fm.ul.pt (N.C.S.)

**Keywords:** flavivirus, circular RNA, RNA structure, virus genomes, circular genomes

## Abstract

The circularization of viral genomes fulfills various functions, from evading host defense mechanisms to promoting specific replication and translation patterns supporting viral proliferation. Here, we describe the genomic structures and associated host factors important for flaviviruses genome circularization and summarize their functional roles. Flaviviruses are relatively small, single-stranded, positive-sense RNA viruses with genomes of approximately 11 kb in length. These genomes contain motifs at their 5′ and 3′ ends, as well as in other regions, that are involved in circularization. These motifs are highly conserved throughout the *Flavivirus* genus and occur both in mature virions and within infected cells. We provide an overview of these sequence motifs and RNA structures involved in circularization, describe their linear and circularized structures, and discuss the proteins that interact with these circular structures and that promote and regulate their formation, aiming to clarify the key features of genome circularization and understand how these affect the flaviviruses life cycle.

## 1. Introduction

Flaviviruses comprise a number of arthropod-borne infections, most of which are prevalent in tropical and subtropical regions around the globe [1,2,3,4]. Prominent members of the *Flavivirus* genus include yellow fever (YFV), tick-borne encephalitis (TBEV), Japanese encephalitis (JEV), Zika (ZIKV), West Nile (WNV), and dengue (DENV) viruses [5,6]. DENV, transmitted by *Aedes* spp. female mosquitoes (*A. albopictus* and *A. aegypti*), is estimated to infect approximately 400 million people per year, with some cases progressing to hemorrhagic dengue fever, leading to over 20,000 deaths worldwide every year [3]. Whereas vaccines are available and effective against YFV, a broadly effective vaccine against all four subtypes of DENV (DENV-1 to DENV-4), or against ZIKV, remains elusive [7,8]. ZIKV, transmitted by the same mosquitoes, is also a global concern due, among other factors, to the continued expansion of these mosquito vectors. Approaches to deal with DENV and ZIKV infections focus mainly on relieving and managing the symptoms. Appropriate medical care of patients progressing to severe dengue can reduce the mortality rate from approximately 20% to 1% [3]. However, this approach alone is insufficient, as medical care may not be affordable or available to vulnerable socio-economic groups in developing countries where flavivirus infections are most prevalent. Therefore, reducing the number of infections is also imperative. Moreover, as highlighted by the ongoing severe acute respiratory syndrome coronavirus 2 (SARS-CoV-2)/COVID-19 pandemic, rapid outbreaks can locally saturate medical care units, even in developed countries, by overwhelming the national healthcare system’s capacity. Further research of preventive or therapeutic strategies is critical, as their immediate availability could mitigate limited medical resources. In addition to accelerated spread, ZIKV poses risks not associated with DENV. Complications such as microcephaly and other neurological conditions arising from ZIKV infection may occur and add to a total mortality rate near 8%, as demonstrated in Brazil [9]. Both DENV and ZIKV cases have been rising in recent years, not only in at-risk developing countries, but also in developed nations, since outbreaks associated with travel are now documented in Europe and North America [3,10,11,12]. Two causal factors, operating concurrently, increase the spread of flavivirus infection: (i) globalization of travel and trade, which escalates virus circulation by exposing naive populations to these infectious agents of foreign origin, plus (ii) the expansion of mosquito habitat resulting from climate change, urbanization, and other factors that help to establish reservoir vector populations in regions that were previously inhospitable to these arthropods [13,14,15,16,17]. These changes in flaviviruses distribution and vector routes support predictions of further expansion worldwide. Thus, effective and readily available treatments and/or prophylactic measures have become a top priority of governments worldwide, with preventative actions being the best approach to deal with the devastating effects of major epidemics on affected populations and their countries’ economies.

## 2. General Genome Structure

**Figure 1:**
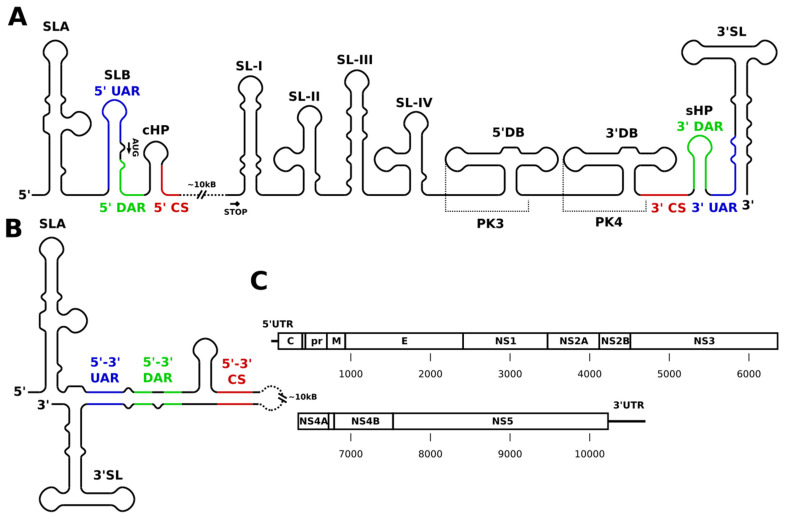
Structural overview of flaviviral genomes. (**A**) Schematic representation of the linear 5′ and 3′ untranslated region (UTRs). Circularization motifs are indicated and labeled in color. The upstream AUG region (UAR; blue) is followed by the downstream AUG region (DAR; green) and the highly conserved circularization sequence (CS; red). Translation initiation happens within stem loop B (SLB) between 5′ UAR and 5′ DAR. The order of circularization sequence motifs is inverted in the 3′ region. (**B**) The circular form of the genome (paired motifs shown in color). SLB, capsid hairpin (cHP), short hairpin (sHP), and 3′SL undergo structural reorganization upon circularization. (**C**) Overview of flaviviral RNA genes. An ≈100 nt 5′ UTR is followed by a ≈10 kb single open reading frame coding a single genome polyprotein, which is post-translationally processed to form the structural (C, prM, and E) and non-structural proteins comprising the flaviviral proteome. The open reading frame is followed by an ≈300–700 nucleotides (depending on species) 3′ UTR containing conserved structural elements.

Flaviviruses contain single-stranded (+)-sense RNA genomes approximately 11 kilobases (kb) in length, with a single open reading frame encoding a genomic polyprotein that is post-translationally processed into its constituent parts. These genomes encode three structural proteins at the 5′ end, namely the capsid (C), precursor membrane, and the envelope protein, plus seven nonstructural proteins (detailed ahead). All flavivirus genomes include a short 5′ untranslated region (UTR) of approximately 100 nucleotides (nt) and a variable-length 3′ UTR containing a number of crucial RNA motifs broadly conserved across the family. As shown in Figure 1A, these motifs comprise a 5′ stem loop (SLA), a short hairpin (SLB), a capsid hairpin (cHP) at the 5′ end, and at the 3′ end of the genome frequently two dumbbell structures (5′ and 3′ DB) upstream of a terminal 3′ stem loop (3′ SL), and another short hairpin (sHP, also referred to as a “small stem loop” or SSL by some authors), which is preceded in sequence by a variable combination of stem-loops (SL), from 1 to 4 (SL-I to SL-IV), at the 5′ end of the 3′ UTR. In addition to these structures in the 5′ and 3′ regions, recent large-scale structure probing and crosslinking studies of DENV and ZIKV revealed a variety of conserved structural elements throughout the coding region, and functional experiments have demonstrated their importance for viral fitness [18,19,20]. In Figure 1, key elements are identified within a simplified graphical linear schematic of flaviviruses genomes. Overall, the flavivirus genome is relatively rich in conserved nucleotide sequences integrated into a variety of functional secondary structure elements with important roles in regulation and viral replication. The following section presents major functional regions individually and describes their particular structural characteristics.

### 2.1. The 3′ UTR

The 3′ UTR (Figure 1) is an approximately 300–700 (depending on species) nucleotide long stretch of RNA terminating in a conserved hydroxylated CU motif, which acts as a recognition site for the viral RNA-dependent RNA polymerase domain of non-structural protein 5 (NS5) [21]. This region includes numerous conserved secondary structures and sequence motifs, each critical to the flavivirus life cycle. Broadly organized, the 3′ UTR is composed of a 5′ variable region (VR), which is dispensable for replication but influences the replication and translation processes, and an essential and relatively invariable 3′ core region, required for genome synthesis [21]. Rigorous structure-based classification divides the 3′ UTR into three discrete domains (the aforementioned core and VR domains, plus an intervening dumbbell region), each with unique roles in viral growth, replication, transcription, translation, and virulence. To better understand their relevance, a brief summary of each domain follows, highlighting their structure and function, with attention given to significant motifs and subregions.

### 2.2. The 3′ Core Region Structures and Sequences Involved in Flavivirus Genome Circularization

Cyclization, defined as the process of circularization that occurs in the flavivirus genome, is discussed in the context of this review. Here, both terms (cyclization and circularization) are used interchangeably. The most conserved 3′ UTR structures and sequences reside in a core region where RNA motifs directly participate in flavivirus genome replication. A single copy of the 3′ conserved sequence (CS1 or 3′ CS) is located upstream of the 3′ stem loop (3′ SL) (Figure 1A). CS1′s 5′ end contains eight consecutive highly conserved nucleotides, known as the 3′ cyclization sequence (3′ CYC), whose corresponding complement is located near the 5′ UTR capsid hairpin (cHP) (described in detail in Section 2.10). This area incorporates the 3′ conserved and cyclization sequences and the less conserved 3′ upstream of AUG region (named 3′ UAR) and 3′ downstream of AUG region (termed 3′ DAR), all of which are involved in genome circularization. CS1 is situated upstream of the 3′ SL, while the 3′ UAR is located within the 3′ SL’s lower stem and the 3′ DAR sits between the 3′ UAR and the 3′ CYC (Figure 1A). The 3′ DAR motif overlaps the short hairpin (sHP) and together with the 3′ UAR form a long-range interaction with complementary sequence motifs in the 5′ UTR. This interaction initiates the protein-independent cyclization of the flavivirus genome.

### 2.3. The 3′ Stem-Loops (3′ SL)

The 3′ terminal stem-loops (3′ SL) (Figure 1A, 1B) were the first RNA structures identified in mosquito-borne flavivirus (MBFV) genomes. Their location at the extreme 3′ end of the flavivirus genome places them directly after the ubiquitous sHP, which is one of two RNA structures found in all flavivirus genomes. 3′ SL structures were validated and are well characterized via biochemical analysis and nuclear magnetic resonance spectroscopy [22,23,24]. In DENV genome 3′ SL consists of 79 nucleotides, while sHP is 14 nucleotides long. The size variation of these motifs is observed in comparison to similar flaviviruses; however, they are representative structures within the genus *Flavivirus*, as there is high sHP sequence conservation throughout the genus as a whole. Mutational and knockout analysis determined the 3′ SL is a *cis*-acting regulatory element essential to virus replication and lethal to infectious flavivirus clones if deleted, but it does not influence translation [25,26]. Likewise, deletion of sHP is lethal in WNV, and loop mutations or base-pairing disruptions in the sHP stem are lethal for WNV and DENV, respectively [27].

Tertiary interactions between the 3′ SL and sHP have been proposed for WNV [28], but mutational analysis and nuclear magnetic resonance spectroscopy were unable to demonstrate this interaction for truncated 3′ WNV RNA [23]. Shi et al. [28] observed a structural transition at low temperatures by circular dichroism, which they attributed to the disorganization of the putative tertiary interaction. However, Davis et al. [23] show that the observed structural change likely stems from the lower portion of the 3′ SL, which contains the UAR element involved in circularization, proposing a role for the host factor eEF1A in the structural rearrangement at this site. This metastable region offers conformational flexibility necessary in the lower portion of the 3′ SL to allow switching from the 3′ SL and 5′ cHP structures to the 3′–5′ RNA–RNA interaction [23]. Through mutational analysis, Davis et al. demonstrated that the eEF1A–RNA interaction is essential for efficient negative-strand synthesis, which is a finding that highlights the multifunctional nature of the 3′ SL [29].

### 2.4. The 3′ Conserved/Cyclization Sequence (CYC) and UAR/DAR

The long-range 3′-5′ RNA–RNA base-pairing interaction of complementary cyclization motifs results in formation of the 5′-3′ panhandle structure, which is a signature of circular flavivirus genomes, and it is a required precursor for negative-strand synthesis. The circular structure initiates the replication of negative-sense genomic progeny RNAs via NS5 C-terminal binding at the last two nucleotides of the 3′ SL. The native sequences of these motifs (3′ CYC and 5′ CYC) are critical to flavivirus replication, as alternative base-pair substitutions were found to reduce virus replication efficiency [30]. Introducing mutations in the CYC adjacent sequence increasingly affected the replication of WNV infectious clones. Five mismatches were lethal, but two or three mismatches reduced replication efficiency and were rapidly reverted [30]. These CYC adjacent sequences function to extend the long-range 3′–’5 RNA–RNA interaction by base-pairing with 5′ UTR complements but is not necessary for genome circularization. The UAR and DAR (Figure 1A, 1B) are poorly conserved between flavivirus species, but 3′–5′ complementarity is preserved within their respective genomes [31]. Mutational experiments demonstrated the sequence requirements of genome cyclization leading to replication where base-pair disruptions in the 3′–5′ CYC formed neither CYC nor UAR interactions, but genome constructs with mismatches in the UAR were able to circularize [32]. Further studies by other groups solved the order in which these 5′–3′ RNA–RNA interactions occur in the flavivirus genome: the 5′ and 3′ CYC sequences initiate the interaction, followed by 5′–3′ DAR annealing, and lastly 5′–3′ UAR pairing to stabilize the complex [31].

### 2.5. Structure Duplication within the 3′ UTR and Flavivirus Host Switching

The 5′ variable region (VR) of the flavivirus 3′ UTR contains two independently folded domains, each comprised of several direct sequence repeats. Studies focusing on duplicated RNA structures derived from these repeats support a model in which the presence of redundant elements allows the virus to accommodate mutations beneficial in one host, but deleterious in the other, thereby increasing the robustness of flavivirus genomes [33]. The flavivirus genome is well adapted to function under disparate environmental stressors, even when changes in sequence compromise replication efficiency. Such an adaptive mechanism may also explain the prevalence of disordered regions throughout the flavivirus proteome [34]. These disordered regions increase the functional diversity of a finite viral proteome by conferring a greater potential for viral–host protein interactions [35].

The dumbbell region (DBR), first identified in the 3′ UTR of DENV genomes, is so named due to one or two tandemly repeated stem-loops with a characteristic dumbbell structure. Initial investigations, spurred by flaviviruses’ curious maintenance of duplicate RNA structures, attempted to explain the biological function of each element. Dumbbell 1 (DB1) and dumbbell 2 (DB2) were initially believed to act primarily as viral replication augmenters [36]. However, secondary structure prediction discovered that these dumbbells occur with unequal frequency in the 3′ UTR of different flavivirus genomes. Additional computational analysis predicted that DBs can also adopt uniquely folded tertiary structure intermediates, via complementary base-pairing between terminal loop 1 and 2 (TL1 and TL2) and their respective pseudoknots (PK3 and PK4), although biochemical data support the existence of only the DB1 TL1–PK3 pseudoknot in DENV2 [24,37]. Further biochemical analysis conducted as cap-independent translation assays revealed that mutations both in TL1 and TL2 reduced DENV2 translation by 60% [37]. The DBR contains two conserved sequences, both overlapping the first loop of their respective structures: repeated conserved sequence 2 (RCS2) belongs to DB1, while conserved sequence 2 (CS2) resides in DB2. Definitive functions related to these two motifs remain unclear, but one study suggests CS2 and RCS2 mutations to adversely impact translation [38]. Overall, DBR is a crucial element in the maintenance of global viral RNA genome structure and plays a major role in translation efficiency.

Despite this information, explicit DBR functions may be challenging to elucidate, as the DB structures, pseudoknots, and RCS2/CS2 sequences all influence genome folding. As discussed elsewhere [39], more recent investigations focus on structure duplication within the DBR and explore the nature of these elements and their significance in flavivirus host switching. Comprehensive sequence analysis of the entire flaviviruses family postulated that duplicated motifs are an outcome of evolutionary pressure favoring viral adaptation to multiple hosts [40]. Recent experimental data confirmed functional roles for duplicate DBR structures in host switching and supports distinct functions for each DB structure. Here, the authors found that each DB structure is under different selective pressure within a particular host [41]. Sequence analysis determined greater DB2 sequence variation within a given MBFV serotype, suggesting that the functional diversification of paralogous DB1 and DB2 occurred after duplication and, therefore, could be driven by host-specific requirements [41]. In support of this hypothesis, the authors found that positively selected mutations occurring in adult mosquito cells that affect the DB2 structure result in enhanced RNA replication [41]. Similarly, the deletion or mutation of SL-II confers a significant replication advantage in mosquito cells, while SL-I mutations have little effect on viral fitness. In contrast, SL-I and SL-II appear to have redundant roles in the human host. Moreover, DENV constructs with either DB1 or DB2 deletion demonstrated opposing effects on luciferase expression, with DB1 deletion yielding a 10-fold decrease and DB2 deletion yielding an 8-fold increase relative to the wild-type virus. These results were confirmed in mosquito cells through measurements of viral RNA accumulation and demonstrate the DBR’s effect on viral titer. In human cells, a double deletion reduced viral replication by nearly 400-fold, while single deletions exhibited the same effect in mosquito cells, albeit to a considerably different degree [41]. As a consequence, viral replication depends on DBR stability as mutations within this region severely affect genome replication efficiency. To the extent that DBR mutations elicit local changes in secondary structure, resulting genome circularization patterns may be altered, explaining the observed phenotypic differences.

### 2.6. The Variable Region (VR)

Spanning approximately 84 nucleotides, the variable region (VR) is situated immediately 3′ of the stop codon and exhibits comparatively low sequence conservation within the 3′ UTR. VR size varies: similar to DBR, the VR may contain one to three conserved direct repeat sequences. In WNV, these are known as conserved and repeated conserved sequence 3 (CS3 and RCS3, respectively). Large sequence alignment analysis suggests that short direct VR repeats are remnants of a larger ancient long repeat sequence [42]. VR length is inconsistent even within virus types, which may contribute to intra-species diversification and aid viral fitness. In DENV and TBEV, the VR exhibits substantial fluctuations in length among serotypes, ranging from less than 50 nucleotides to more than 120 [43,44]. In TBEV strains passaged in mammalian cells or isolated from human patients, these size fluctuations arise from sequence deletions or poly-A insertions [45,46,47]. Such length alterations, particularly direct repeats, correlated to RNA replication, suggest a mechanism for flavivirus host cell adaptation and may explain virulence in severe clinical cases [48]. Structurally, this subregion contains species-specific stem loops of varying size and number, some of which with functions crucial to flavivirus fitness. Efficient genome replication, but not translation or infectious viral particle production for DENV-1/2 in mammalian cells, requires complete and intact VR sequences [36,49]. Furthermore, detailed studies of the VR have uncovered subregion specialization that may endow members of the *Flavivirius* genus with the ability to calibrate viral RNA accumulation in response to host cell type during infection. Based on local sequence conservation, the VR can be divided into 5′ hypervariable (HVR) and 3′ semi-variable (SVR) regions. In 2007, Tajima et al. reported that deletions occurring within either or both subregions reduced the growth of recombinant DENV-1 virions in mammalian cells, but reverse sequence alterations in the SVR alone were able to influence viral replication in host cells, but not in mosquito cells [36,49]. Whereas HVR supports efficient DENV-1 growth in a sequence-independent manner and reversed SVR sequences impeded DENV-1 growth kinetics, it follows that the length of the HVR, along with some critical SVR secondary structure, is important for adequate DENV-1 replication.

In addition to genome replication, evidence from studies in mouse models suggests the VR is also a critical flavivirus virulence factor. Using chimeric TBEV constructs derived from strains of disparate pathogenicity, a highly pathogenic Sofjin-HO Far-Eastern subtype VR was transposed with the VR from a low pathogenic strain (Oshima 5–10) [50]. Oshima-derived chimeric viruses with Sofjin VR achieved virulence levels comparable to native Sofjin viruses. Furthermore, the nearly identical viral titer levels of chimeric Oshima resulted in histopathological changes to brain tissue that are characteristic of Sofjin infection. The following studies suggested that TBEV pathogenicity is associated with VR conformational structure [50,51], as recombinant TBEV constructs with specific VR stem-loop deletions displayed increased virulence in mouse brain tissue, with no reduction in subgenomic flavivirus RNA (sfRNA) production. Overall, the VR acts as a modulator of genome replication as well as host and cell type selectivity, and it impacts the virulence of individual strains or serotypes [33].

VR stem-loops vary considerably both in size and number between flavivirus species; the stem-loops of the WNV VR are the most well-characterized, comprising four stem-loops, designated SL-I through SL-IV. SL-II and SL-IV of WNV may provide resistance to host nuclease activity [51]. SL-II, SL-IV, and (potentially) DB1 are capable of stopping genome degradation by pausing exoribonuclease Xrn1 at VR stem-loops. Pausing results in the accumulation of undigested sfRNA fragments [52,53]. These non-coding fragments help neutralize the antiviral responses of mosquito and human cells in a concentration-dependent manner [54]. Undigested sfRNA fragments achieve this outcome via two distinct mechanisms: (i) antagonization of the host’s innate immune response by blocking interferon-α activity, and (ii) direct inhibition of the exoribonuclease Xrn1 activity at stem-loop sites [55,56]. sfRNA production via this mechanism has been shown in human and mosquito cells for ZIKV [57]. Additionally, duplicate DENV structures SL-I and SL-II are functional analogues of WNV SL-II and SL-IV and are composed of nearly identical sequence repeats. These VR stem-loops also form pseudoknots through interactions between apical loop sequences and conserved pseudoknots located in their respective basal stems. The presence of consecutive pseudoknots may act as size-restricting checkpoints during the production of biologically active sfRNA fragments and promote their formation. These functions in concert may help to protect other viral genome RNAs from degradation. The structure of the sfRNA of Murray Valley encephalitis virus was determined by crystallization and demonstrates resistance to Xrn1-mediated degradation [58].

VR stem-loops formed from direct repeat sequences (e.g., DENV SL-II) garner mutations that can provide host-specific adaptations. One study revealed a hot spot for sequence variations in the 3′ UTR in DENV populations restricted to replicate in either mosquito or human cells [33]. Subsequent deep sequencing discovered that mutations selected for in mosquito cells mapped to SL-II. The authors observed that mutations disrupting the SL-II structure increased viral fitness in mosquito cells relative to DENV constructs harboring native SL-II [33]. Conversely, SL-II disrupting mutations reduced viral replication in human cells [33]. SL-I does not induce this effect in mosquito cells and remains intact during flavivirus replication in both mosquito and human cells. These disparate observations support the functional diversification hypothesis of duplicated VR elements.

### 2.7. The 5′ UTR

The flavivirus genome begins with the approximately 100 nucleotide long, highly conserved, 5′ UTR (Figure 1). In relation to the 3′ UTR, the shorter length of 5′ UTR exemplifies the region’s well-defined structural landscape and concise range of functions. The 5′ terminus is decorated with an m7GpppAmpN2 type I cap structure consisting of N7 and 2′OH methyl groups that is followed by a conserved AG dinucleotide [59]. The 5′ UTR contains one and a half RNA stem-loops that act as distinct functional domains during viral genome synthesis and translation. These structures include the large highly conserved stem-loop A (SLA), a short relatively variable stem-loop B (SLB), and the downstream cHP segment. The following is a brief survey of each structure and its primary functions.

### 2.8. The Stem-Loop A (SLA)

The roughly 70-nucleotide long SLA (Figure 1A, 1B) is the second of two RNA elements preserved throughout the flaviviruses family (the other one being sHP, which was already discussed above in Section 2.3.). Its highly conserved Y-shaped secondary structure consists of a main stem-loop and smaller side stem-loop. SLA size and sequence vary throughout the flavivirus genus (Figure 1A). SLA acts as a promoter of negative-strand RNA synthesis and interacts with NS5 MTase during capping. Interactions between the NS5 methyltransferase (MTase) domain and specific architectural features of the SLA (the internal loop, as well as basal and upper stem residues) position the NS5 RNA-dependent RNA polymerase domain near the 3′ end of the genome during the 3′–5′ RNA–RNA long-distance interaction. At this point, RNA-dependent RNA polymerase activity can initiate de novo synthesis of the negative strand [60,61]. SLA also directs the addition of a 5′ cap during synthesis by repositioning the 5′ end of the nascent genomic transcript near the MTase active site for catalytic addition of the guanylyl and methyl groups [62,63]. This is a critical function, as translation of the mosquito-borne flaviviruses’ polyprotein is modulated in a cap-dependent manner [64]. Thus, the SLA region contributes to regulation and fine-tuning of long-ranged interactions, as well as to the production of viral genome copies, by interacting with NS5 MTase, leading to the proper placement of NS5 RNA polymerase domain.

### 2.9. The Stem-Loop B (SLB)

A small stem-loop present in most flavivirus species, known as stem-loop B (SLB) (Figure 1A,B), resides downstream of the 5′ SLA. DENV SLA and SLB are separated by a flexible oligo (U) tract that promotes structural rearrangement during linear and circular genome cycling [65]. SLB exhibits greater size and structural variability than SLA and also contains the AUG translation initiation site as part of its stem region. The 5′ UAR sequence overlaps SLB in both WNV and DENV, while the 5′ DAR sits in SLB and/or in the adjacent 3′ sequence (Figure 1A). The placement of these long-range RNA–RNA interaction motifs requires the entire SLB to unfold during genome circularization, whereupon hybridization of the former stem-loop region with its 3′ complement closes the circular structure (Figure 1B). Compelled by this mechanism, SLB participates in long-range RNA–RNA interactions that form canonical flavivirus circularization patterns. Furthermore, SLB’s 3′ flanking region acts as an additional recruitment site for NS5, the flaviviral RNA-dependent RNA polymerase (RdRp) [66]. Liu and Qin [66] provide a detailed discussion of cis-acting flavivirus structures, some of which have recently been characterized by nuclear magnetic resonance spectroscopy [67]. These data show high structural conservation between DENV and WNV SLB structures and confirm SLB’s role as a key *cis*-acting element involved in circularization. The lower stem of SLB is a U-rich region known as the 5′-UAR-flanking stem (UFS). Here, base-pair identity acts to lock 5′UAR/SLB conformation, which is an essential recognition structure for NS5 recruitment and, also, a switching mechanism for viral RNA synthesis [66]. These competing functions derive from UFS helix stability. Primarily consisting of destabilizing U·A/A·U base pairs, the UFS duplex is likely more flexible than SLB’s upper stem-loop [68]. Neutral energetic contributions of G·C/C·G substitutions allow base-stacking interactions to dominate UFS stability resulting in decreased genome cyclization and viral RNA replication [66,68]. Functioning in tandem, the interplay between SLB structure and UFS stability may be a critical determinant for flavivirus cyclization and fitness, with further investigation being required.

### 2.10. The Capsid Hairpin (cHP)

The cHP is a stable and well-conserved hairpin that follows downstream of SLB and covers the first nucleotides of DENV and WNV capsid coding regions (Figure 1A). Along with the 3′ sHP, the 5′ cHP is only present in the linear flavivirus genome, as some of their stem nucleotides participate in alternative long-distance base pairing during cyclization. The cHP regulates the selection of the translation initiation codon by positioning a host ribosome near the first AUG start codon of SLB. The efficiency with which the cHP is able to direct translation initiation from the suboptimal first start codon is independent of its position and its sequence, but is instead proportional to its thermodynamic stability [69]. Stable stem-loop structures existing downstream of an AUG codon that is integrated in a poor Kozak context can pause translation machinery such that it must first unwind the hairpin before proceeding [70]. Through this stalling mechanism, the cHP acts as a translation enhancer by facilitating extended ribosomal contact with the optimal flavivirus start codon.

The cHP also acts as *cis*-replicating element in both WNV and DENV genomes, distinguishing it as an RNA domain with multifunctional influence over the flavivirus life cycle [71]. Translation initiation is promoted during early infection when the viral genome has not acquired the circular conformation required for replication. In the linear genome, the cHP stem-loop causes the ribosomal complex to linger briefly at the appropriate start codon, thereby encouraging its recognition. When switching to genome replication, long-range RNA–RNA interactions are established between the 5′ and 3′ genome ends to form the circular flavivirus genome. Induction of the circular conformation shortens the cHP stem, which in turn lengthens the neighboring 3′ stretch of RNA. Therefore, structural rearrangement of the translation competent scaffold exposes the 5′ CS that overlaps the 3′ component of the cHP stem and helps organize the replication competent circular genome (Figure 1A,B) [71]. Thus, not only SLB, but also the cHP have a key influence over circularization.

## 3. Circularization Structures

The occurrence of 5′-3′ circularization through intramolecular duplexes formation is conserved across the flaviviruses family, and it is effected by three complementary regions: (i) the DAR, (ii) the UAR, and (iii) the circularization sequence (CS) [72,73]. The circularization motif was first identified as a conserved sequence near the 3′ UTR by Hahn et al. [74]. These authors also located a complementary conserved element in the 5′ UTR and postulated potential circularization in a flaviviruses. The functional importance of these sites was shown by Men et al. [75] by examining various deletion mutants in the 3′ UTR [74]. Their study showed that all 3′ UTR deletion mutants of DENV-4 were viable (albeit attenuated), as long as the deletion did not include the last 113 nucleotides containing the circularization motif and 3′ SL. Subsequently, 5′–3′ interactions were shown to be important for other flaviviruses [76,77,78]. The DAR/UAR/CS cyclization motif is thought to act as a single regulatory unit. As Zhang et al. [79] have shown, deletion of CS in WNV is lethal, but can be rescued by compensatory strengthening of interactions in the DAR/UAR regions.

The UAR is located closer to the 5′ and 3′ ends than the CS. The UAR and DAR are mostly contained in structured elements in the linear form of the genomic RNA, whereas the CS is located in a single-stranded stretch between 3′ DB and sHP. Consequently, these structural elements undergo rearrangement upon circularization. Such a feature has been previously observed in different (+)-sense RNA viruses. Olsthoorn et al. described a conformational change in the 3′ region of plant viruses of the Alfamovirus and Ilarvirus families that is necessary to initiate viral replication [80]. In particular, 5′-3′ circularization as a regulatory point controlling RNA replication has been described for Tombusvirus, as negative-strand synthesis is inhibited by formation of the circular form [81]. Circularization as a regulatory mechanism is not confined to the 5′ and 3′ terminal regions, as demonstrated by Zhang et al. [82,83]. They found that an interaction of a 3′ structural element in the turnip crinkle virus genome with a large internal loop structure suppresses negative-strand RNA transcription and posit that such inhibitory motifs are responsible for asymmetric ratios of negative to positive strands in a range from 1:10 to 1:1000 during RNA-dependent RNA polymerase-dependent transcription [82,83].

Circularization of the genome is essential for its replication [76,77,78,84]. Corver et al. [84] identified specific interacting nucleotides of YFV in the 5′ UTR necessary for replication, such as an 18 nt stretch at positions 146–163, with a slightly longer stretch of 21 nucleotides (146–166), required for full replication efficiency, which is a longer segment than the universally conserved 8 nucleotides across flaviviruses. Noteworthy, not all sequences involved in the 3′–5′ interaction are located in the 5′ UTR (as some are in the coding region). Circularization has been shown to be necessary for (-) strand synthesis. Moreover, requirements for replication rely on the presence of specific nucleotides at certain positions beyond the requirement for complementarity [85]. Alvarez et al. [85] demonstrated that specific mutations within the UAR caused a significant delay in viral replication for variants with multiple mutations, despite reconstituted complementarity and the ability to form cyclical genomes in these variants. A possible explanation for this effect is that the sequences are multifunctional and have specific roles in the linear and circular form, forming either a local or a long-range interaction; whereas the transposition of these sequences would maintain their function in the circular state, the function in the local context of the linear form would be disrupted [85].

Lott and Doran [86] suggested that under cellular conditions, the formation of dimeric or multimeric forms connected by their respective circularization sequences is more likely. This argument is based on molecular crowding in local environments that arise from the remodeling of the endoplasmic reticulum membrane induced by flavivirus infection [87]. Brinton et al. [88] dispute that the formation of concatemers on the ground would lead to increased efficiency of minus strand synthesis, which is inconsistent with available evidence on minus strand abundance throughout the replication cycle [88]. WNV variants with a high abundance of minus strand have been shown to have decreased virus production and decreased positive strand levels [29]. Evidence from structure-probing studies show that a cyclized form is present in virions [18,19,20] and is consistently one of the most pronounced signals.

### 3.1. Internal Circularization

**Figure 2:**
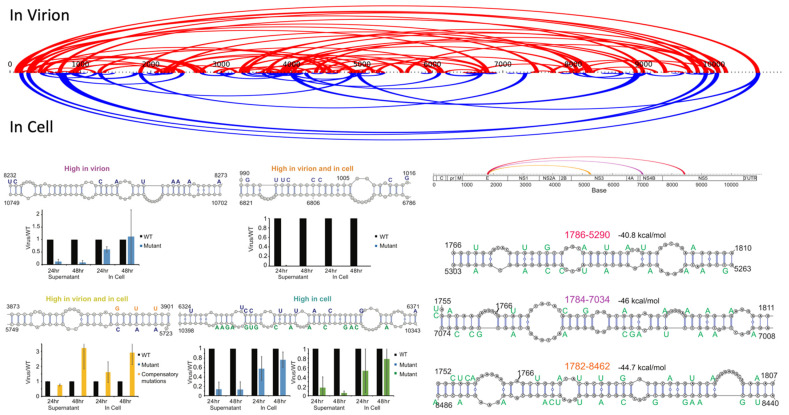
Internal circularization sites of DENV-1 genome, as determined by SPLASH cross-linking [19]. It is apparent that the number of internal circularization motifs in virions (red) is much higher than those identified in infected cells. A number of interactions persist in cells (blue). This may be attributed to the spatial constraints imposed by the virion shell or it may be a prerequisite for packaging. Functional assays demonstrate the importance of these motifs for viral fitness, with structure-disrupting changes causing a pronounced drop in viral activity. Compensatory mutations restore viral fitness. Several of the identified internal circularization sites show multiple interaction partners in the SPLASH experiment and structure models of the relevant regions show competing base pairs among the possible partners. It is currently unclear whether these competing interactions form in a stochastic manner or whether specific interactions are present at different stages of the viral life cycle and/or rearrange dynamically.

In addition to 5′-3′ circularization, recent flaviviruses studies revealed internal long-range interactions of functional relevance. These motifs involve the flaviviral genome-coding region and form persistent long-range interactions. Some interactions are exclusive to the packaged state of the genome within virions, whereas others are found in the virion and within infected cells. Chemical cross-linking techniques used to reveal these interactions include COMRADES (cross-linking of matched RNAs and deep sequencing) [18] and SPLASH (sequencing of psoralen crosslinked, ligated, and selected hybrids) [19,20]. An overview of internal circularization sites of DENV genome is depicted in Figure 2.

The functional role of these internal circularization motifs is particularly interesting as the associated sequence elements are subject to two distinct sources of evolutionary constraint: when sequences that include a coding region change their nucleotides and evolve, the respective translated protein regions must maintain protein function while simultaneously maintaining a functional level of the interactions that are promoted by the nucleotide sequence. In addition, a nucleotide-level constraint exists to maintain RNA complementarity. DENV was particularly suitable to study these interactions, as it has four distinct serotypes that have been surveilled throughout lengthy periods of time and wide geographical areas. This has enabled the gain of a relatively comprehensive collection of viral sequences that lend themselves to statistical analysis for covariation. Covariation in genome sequences allows us to identify evolutionary constrained base pairs in genome structures. The basic approach behind this analysis is to assess complementary mutations for variation at each position. If the maintenance of a base pair is functionally important, then mutation in a specific base will induce a compensatory mutation at the paired base with higher than expected likelihood, which can be statistically evaluated. This statistical evaluation of covariation is implemented in the R-scape tool [89]. The key limitation to this methodology for structure identification is that it requires a sufficient stock of genetic history of the sequence under investigation. Dengue is a rare case where the breadth and depth of sequences available allow for such an analysis. We attempted to apply the same technique to study ZIKV, but we were severely limited due to the lack of diversity in available sequences. Most ZIKV sequences were deposited during the 2015–2016 Zika pandemic and, hence, are of limited genetic variability, making it challenging to identify covarying base pairs.

Previous studies of internal circularization motifs have revealed competing structures within the genome of flaviviruses [19]. Only about two-thirds of interactions identified by cross-linking have unique interaction partners, and a number of sites show highly promiscuous behavior with nine or more interaction sites [19]. It cannot be ruled out that some of these interactions are artifacts of non-specific crosslinking stemming from the experimental protocol, but structural modeling of the proposed interactions reveals likely circular structures for multiple partner sites for some sequences [19]. A caveat of chemical crosslinking procedures is that the position resolution is limited by the length necessary for unique read mapping and is therefore usually in the tens of nucleotides. As structural motifs may be both adjacent and short, it is possible that these ostensibly competing interactions coexist in close spatial proximity. However, structural models again suggest that the actual bases involved are shared between multiple predicted interactions and, hence, true competition for specific sites exists in flavivirus genomes [18,19,20].

The functional relevance of these internal circularizing interactions has been established by mutational studies. These show severe attenuation of viral fitness even if preserving both the protein sequence and avoiding rare codons that might reduce translation efficiency. Moreover, compensatory mutations at partner sites were able to restore viral fitness, hence demonstrating convincingly that the structure at the RNA level is indeed functionally necessary. The precise function of these structural motifs in the coding sequence remains elusive. Several possible explanations for their biological importance exist. One hypothesis is that these structural motifs promote viral packaging into nascent virions by compacting the genome after replication. In a similar vein, these structural motifs, together with other shorter-range structures, may form anchor points for host or viral protein interactions involved in viral replication and/or packaging. Unfortunately, current experimental data are not able to answer whether these motifs are of a dynamic or static nature. Evidence of competing interactions suggest that alternate conformations are possible but, at present, it is unclear whether these interactions will: (i) be formed in a “thermodynamic funnel”, where the genome folds into a local minimum and remains in a specific conformation until disturbed (e.g., by translation or replication); or, (ii) whether these motifs are dynamic and can rearrange in response to environmental conditions, possibly performing a regulatory function.

### 3.2. Protein Interactions

**Figure 3:**
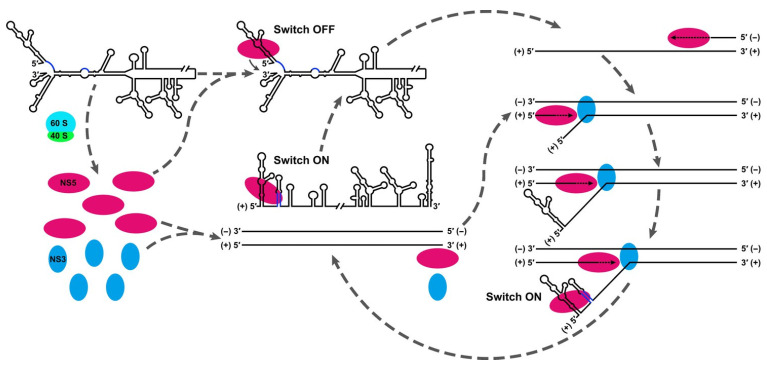
Schematic model elucidating the functional mechanism of the UAR-flanking stem (UFS) switch. Genome cyclization is crucial for the non-structural protein 5 (NS5) translocation to the RNA synthesis initiation site. Adapted with permission from [66].

The importance of the circularization motif for self-primed genome replication has been demonstrated by You et al. [90,91]. They also posit that several cellular proteins previously identified to interact with this motif [92,93,94] could play a role in modulating the stability of the circularized structure. Interestingly, Shi et al. [92] were able to find proteins specifically interacting with the (-)-strand of the 3′ stem-loop structure. Blackwell and Brinton [93] identified the key translation elongation factor 1 alpha as a crucial host protein interacting with the 3′ stem loop. This protein has been identified as a key host factor in the replication of diverse viruses, including retroviruses, flaviviruses, and bacteriophages [95]. Concerning the non-structural viral proteins, NS5 is, in this context, a key protein, with a major role in regulating cyclization and viral activity, as presented in Figure 3 [66]. NS5 has methyltransferase, guanylyltransferase, and RNA-dependent RNA polymerase (RdRp) activities. This enables NS5 to interact and bind to the flavivirus RNA, to other viral nonstructural proteins, and to host factors. Filomatori et al. [96] showed that the NS5 methyltransferase domain interacts specifically with SLA in the 5′ region, which through the circularization motif brings NS5 into proximity with the 3′ transcription initiation side. Subsequently, Lodeiro et al. [97] proceeded to identify the specific regions of SLA responsible for polymerase binding and identified the crucial role of the apical loop and side stem loop regions. These regions are conserved across flaviviruses. Dong et al. [98] investigated the potential of the methyltransferase domain of NS5 as an antiviral target, but they mainly focused on the methylation activity of this domain. Overall, it is crucial for assembly of the viral replication complex essential for vRNA synthesis [66]. Most importantly, it regulates cyclization and viral synthesis by binding to the stem loop region. Moreover, integrity of the 3′ stem loop is ensured by the nucleotidyl transferase activity of NS5, as Teramoto et al. [99] have demonstrated. Deletions (e.g., caused by RNase digestion of the 3′ end) cause a severe drop in viral fitness, as measured by NS1 antigen production. However, reconstitution of the deletion mediated by NS5 does occur and recovers the original sequences as consensus, enabling the reformation of the 3′ stem loop structure [99]. Another mechanism of initiation site conservation was described by *Selisko* et al., [100] who demonstrated that NS5 polymerase forms and/or elongates pppAG dinucleotides, even in the case of incorrect 3′ ends, in the presence of Mg^2+^ ions [100].

The C protein plays a key role in the formation of the circular form of the RNA genome. Ivanyi-Nagy et al. [101] demonstrated that the C protein is an important RNA chaperone, and that the interaction of the C-terminal region of the C protein substantially increases the rate of 5′-3′ circularization [102]. The C protein has N-terminal and C-terminal RNA-binding regions [103], from which only the C-terminal region acts as a chaperone [101]. In cells, the C protein may be supplemented in this function by the abundance of host RNA chaperones [104]. In addition to the chaperone activity of the C protein, the weak chaperone activity of NS5 has been described by Pong et al. [105], who concurrently could not identify any chaperone activity of NS3. The heterogeneous nuclear ribonucleoprotein A2 (hnRNP A2) was identified as an important host factor interacting with the C protein, NS5, and the 5′ UTR of the minus strand of JEV [106]. The precise functional nature of these interactions in the context of flaviviruses remains unclear. hnRNP interactions are proliferative (e.g., in Sindbis virus and enteroviruses), while exerting an antiviral effect (e.g., in hepatitis C virus). It is presumed that this is associated with hnRNPs effect on viral and host gene expression [107]. NS3 has been shown to promote complementary strand annealing, both internal to the viral genome and between host and viral RNA [108]. Moreover, Gebhard et al. [108] showed that while NS3 promotes annealing in an ATP-independent manner, increased ATP concentration allows NS3 to unwind RNA. Recently, Swarbrick et al. [109] have shown that the interaction between the 5′ end of flavivirus RNA and NS3 is sequence-specific, with guanosines in the 2 and 5 positions causing significantly higher activity.

Bidet et al. [110] provided a summary of a variety of host factors positively identified to interact with flavivirus RNA, either promoting or inhibiting viral proliferation. It is notable that most of the identified host factors interact with the 3′ stem loop, 3′ dumbbell, or the 5′ and 3′ UTRs involved in genome circularization. However, it is unclear if this reflects the balance of interactions inside cells or if it is an artifact of the level of research applied to these specific regions, while for instance the coding region has been less explored. Most of the proteins summarized by Bidet et al. are host factors generally involved in the transcription and processing of RNA [110]. A key host factor interacting with both 5′ and 3′ ends simultaneously is the host protein La. Initially, La has been shown to bind the 3′ stem loop structure by Vashist et al. [111], who subsequently demonstrated high affinity of the 5′ UTR of JEV to La as well [112]. Such an interaction pattern by La protein has also been shown for DENV4 [113], indicating that this is a shared mechanism across flaviviruses. They posit that this simultaneous interaction may promote circularization and thereby enhance viral replication. Chien et al. [114] demonstrated that FUSE binding protein 1 (FBP1) also interacts with both 5′ and 3′ UTRs in JEV, but they found that the overexpression of FBP1 induced a reduction in viral replication and the knockdown of FBP1 promoted enhanced replication, which points to a different mechanism of action than La protein for this antiviral interaction. The degradation of viral RNA is an important host defense mechanism. In the context of flaviviral RNA, the ribonuclease MCPIP1 was shown to broadly reduce viral activity in cells [115]. The above-mentioned undigested sfRNA fragments [52,53] that reduce antiviral responses by inhibiting exoribonuclease Xrn1 activity at stem-loop sites [55,56] are also an example of key interactions of RNA with the host proteins, in this case aiming at protecting nascent viral RNA copies from degradation.

## 4. Concluding Remarks

Viruses have evolved several mechanisms in order to increase their protection against host cellular surveillance and attack mechanisms, as well as to manipulate cellular pathways to their own benefit, subsequently increasing their survival and persistence in the host. Genome circularization is one such mechanism that has been reported in a broad range of viruses. *Flavivirus*, which include several human pathogens, is one of the best-described genera exhibiting genome circularization and long-range RNA–RNA interactions (internal circularization). Several sequences have been identified as essential for these processes. It was demonstrated that genome circularization is a regulatory mechanism essential for the synthesis of the negative strand during genome replication. Studies in which long-range RNA–RNA interactions were impaired have shown drastic decreases in viral fitness, suggesting their importance in the assembly and encapsidation (packaging) of the new virions. It is also proposed that these interactions generate secondary and tertiary structures that act as anchor points for viral and/or cell proteins that facilitate virion packaging. Both mechanisms provide ideal conditions for viral life cycle steps. Several host proteins have been identified as essential to the circularization process and necessary for stabilization of the circular form (e.g., hnRNP). Viral proteins also participate in circularization, such as the C protein (RNA chaperone), which increases the genome circularization rate.

The flavivirus capsid protein is proposed to bind the RNA genome and promote its packing within the tight space of the virion. This function would not be possible without a concomitant condensing of the genome via circularization and long-range interactions. Mechanistically, specific loops, hairpins, and tertiary structure elements may coordinate RNA binding to the protein in addition to specific sequence motifs. However, the absence of circularization and long-range interactions would make viral genome packing extremely difficult. This is supported by the observation that such interactions are seen in genomes of other viruses. For example, long-range RNA–RNA interactions have also been reported for coronaviruses, being involved in essential steps of the viral life cycle. It is possible that if flaviviruses evolved to infect a single host, circularization and evolution into a latent virus form might have occurred. However, since flaviviruses infect several hosts, and given that several regions involved in circularization are also involved in virulence and host adaptability, it follows that it is more difficult for a virus that switches between hosts to develop and evolve into a fully circular latent genome form. Guided by these limitations, flaviviruses take advantage of circularization and long-range RNA–RNA interactions. While interesting from an evolutionary perspective, this knowledge is also relevant in the medical context. This is clear evidence that circularization is extremely important to the flavivirus life cycle and is therefore an interesting target that should be thoroughly explored for therapies against flaviviruses.

## Data Availability

Data sharing not applicable.

## References

[1] Aedes Albopictus - actsheet for Experts. https://www.ecdc.europa.eu/en/disease-vectors/facts/mosquito-factsheets/aedes-albopictus.

[2] Aedes Aegypti - Factsheet for Experts. https://www.ecdc.europa.eu/en/disease-vectors/facts/mosquito-factsheets/aedes-aegypti.

[3] Dengue and Severe Dengue. https://www.who.int/news-room/fact-sheets/detail/dengue-and-severe-dengue.

[4] Zika Virus. https://www.who.int/news-room/fact-sheets/detail/zika-virus.

[5] Mukhopadhyay S., Kuhn R.J., Rossmann M.G. (2005). A structural perspective of the Flavivirus life cycle. Nat. Rev. Microbiol..

[6] Ng W.C., Soto-Acosta R., Bradrick S.S., Garcia-Blanco M.A., Ooi E.E. (2017). The 5′ and 3′ untranslated regions of the flaviviral genome. Viruses.

[7] Thomas S.J., Yoon I.K. (2019). A review of Dengvaxia^®^: Development to deployment. Hum. Vaccines Immunother..

[8] Halstead S. (2019). Recent advances in understanding dengue. F1000Research.

[9] Da Cunha A.J.L.A., De Magalhães-Barbosa M.C., Lima-Setta F., De Andrade Medronho R., Prata-Barbosa A. (2016). Microcephaly case fatality rate associated with Zika virus infection in Brazil. Pediatr. Infect. Dis. J..

[10] Dengue in the US States and Territories | Dengue | CDC. https://www.cdc.gov/dengue/areaswithrisk/in-the-us.html.

[11] Statistics and Maps | Zika Virus | CDC. https://www.cdc.gov/zika/reporting/index.html.

[12] Surveillance Atlas of Infectious Diseases. https://atlas.ecdc.europa.eu/public/index.aspx?Dataset=27&HealthTopic=70.

[13] Iwamura T., Guzman-Holst A., Murray K.A. (2020). Accelerating invasion potential of disease vector Aedes aegypti under climate change. Nat. Commun..

[14] Kamal M., Kenawy M.A., Rady M.H., Khaled A.S., Samy A.M. (2018). Mapping the global potential distributions of two arboviral vectors Aedes aegypti and Ae. albopictus under changing climate. PLoS ONE.

[15] Caminade C., Medlock J.M., Ducheyne E., McIntyre K.M., Leach S., Baylis M., Morse A.P. (2012). Suitability of European climate for the Asian tiger mosquito Aedes albopictus: Recent trends and future scenarios. J. R. Soc. Interface.

[16] Powell J.R., Tabachnick W.J. (2013). History of domestication and spread of Aedes aegypti—A review. Mem. Inst. Oswaldo Cruz.

[17] Kraemer M.U.G., Reiner R.C., Brady O.J., Messina J.P., Gilbert M., Pigott D.M., Yi D., Johnson K., Earl L., Marczak L.B. (2019). Past and future spread of the arbovirus vectors Aedes aegypti and Aedes albopictus. Nat. Microbiol..

[18] Ziv O., Gabryelska M.M., Lun A.T.L., Gebert L.F.R., Sheu-Gruttadauria J., Meredith L.W., Liu Z.-Y., Kwok C.K., Qin C.-F., MacRae I.J. (2018). COMRADES determines in vivo RNA structures and interactions. Nat. Methods.

[19] Huber R.G., Lim X.N., Ng W.C., Sim A.Y.L., Poh H.X., Shen Y., Lim S.Y., Sundstrom K.B., Sun X., Aw J.G. (2019). Structure mapping of dengue and Zika viruses reveals functional long-range interactions. Nat. Commun..

[20] Li P., Wei Y., Mei M., Tang L., Sun L., Huang W., Zhou J., Zou C., Zhang S., Qin C.-F. (2018). Integrative Analysis of Zika Virus Genome RNA Structure Reveals Critical Determinants of Viral Infectivity. Cell Host Microbe.

[21] El Sahili A., Lescar J. (2017). Dengue virus non-structural protein 5. Viruses.

[22] Brinton M.A., Fernandez A.V., Dispoto J.H. (1986). The 3′-nucleotides of flavivirus genomic RNA form a conserved secondary structure. Virology.

[23] Davis W.G., Basu M., Elrod E.J., Germann M.W., Brinton M.A. (2013). Identification of cis-Acting Nucleotides and a Structural Feature in West Nile Virus 3′-Terminus RNA That Facilitate Viral Minus Strand RNA Synthesis. J. Virol..

[24] Sztuba-Solinska J., Teramoto T., Rausch J.W., Shapiro B.A., Padmanabhan R., Le Grice S.F.J. (2013). Structural complexity of Dengue virus untranslated regions: Cis-acting RNA motifs and pseudoknot interactions modulating functionality of the viral genome. Nucleic Acids Res..

[25] Elghonemy S., Davis W.G., Brinton M.A. (2005). The majority of the nucleotides in the top loop of the genomic 3′ terminal stem loop structure are cis-acting in a West Nile virus infectious clone. Virology.

[26] Tilgner M., Deas T.S., Shi P.Y. (2005). The flavivirus-conserved penta-nucleotide in the 3′ stem-loop of the West Nile virus genome requires a specific sequence and structure for RNA synthesis, but not for viral translation. Virology.

[27] Villordo S.M., Gamarnik A.V. (2013). Differential RNA Sequence Requirement for Dengue Virus Replication in Mosquito and Mammalian Cells. J. Virol..

[28] Shi P.Y., Brinton M.A., Veal J.M., Zhong Y.Y., Wilson W.D. (1996). Evidence for the existence of a pseudoknot structure at the 3′ terminus of the flavivirus genomic RNA. Biochemistry.

[29] Davis W.G., Blackwell J.L., Shi P.-Y., Brinton M.A. (2007). Interaction between the Cellular Protein eEF1A and the 3′-Terminal Stem-Loop of West Nile Virus Genomic RNA Facilitates Viral Minus-Strand RNA Synthesis. J. Virol..

[30] Basu M., Brinton M.A. (2011). West Nile virus (WNV) genome RNAs with up to three adjacent mutations that disrupt long distance 5′-3′ cyclization sequence basepairs are viable. Virology.

[31] Friebe P., Shi P.-Y., Harris E. (2011). The 5′ and 3′ Downstream AUG Region Elements Are Required for Mosquito-Borne Flavivirus RNA Replication. J. Virol..

[32] Polacek C., Foley J.E., Harris E. (2009). Conformational Changes in the Solution Structure of the Dengue Virus 5′ End in the Presence and Absence of the 3′ Untranslated Region. J. Virol..

[33] Villordo S.M., Filomatori C.V., Sánchez-Vargas I., Blair C.D., Gamarnik A.V. (2015). Dengue Virus RNA Structure Specialization Facilitates Host Adaptation. PLoS Pathog..

[34] Martins I.C., Santos N.C. (2020). Intrinsically disordered protein domains in flavivirus infection. Arch. Biochem. Biophys..

[35] Giri R., Kumar D., Sharma N., Uversky V.N. (2016). Intrinsically Disordered Side of the Zika Virus Proteome. Front. Cell. Infect. Microbiol..

[36] Alvarez D.E., De Lella Ezcurra A.L., Fucito S., Gamarnik A.V. (2005). Role of RNA structures present at the 3′UTR of dengue virus on translation, RNA synthesis, and viral replication. Virology.

[37] Manzano M., Reichert E.D., Polo S., Falgout B., Kasprzak W., Shapiro B.A., Padmanabhan R. (2011). Identification of cis-acting elements in the 3′-untranslated region of the dengue virus type 2 RNA that modulate translation and replication. J. Biol. Chem..

[38] Wei Y., Qin C., Jiang T., Li X., Zhao H., Liu Z., Deng Y., Liu R., Chen S., Yu M. (2009). Translational regulation by the 3′ untranslated region of the dengue type 2 virus genome. Am. J. Trop. Med. Hyg..

[39] Villordo S.M., Carballeda J.M., Filomatori C.V., Gamarnik A.V. (2016). RNA Structure Duplications and Flavivirus Host Adaptation. Trends Microbiol..

[40] Gritsun T.S., Gould E.A. (2006). Origin and Evolution of 3′Utr of Flaviviruses: Long Direct Repeats as A Basis for the Formation of Secondary Structures and Their Significance for Virus Transmission. Adv. Virus Res..

[41] de Borba L., Villordo S.M., Marsico F.L., Carballeda J.M., Filomatori C.V., Gebhard L.G., Pallarés H.M., Lequime S., Lambrechts L., Vargas I.S. (2019). RNA structure duplication in the dengue virus 3′ UTR: Redundancy or host specificity?. MBio.

[42] Gritsun D.J., Jones I.M., Gould E.A., Gritsun T.S. (2014). Molecular archaeology of Flaviviridae untranslated regions: Duplicated RNA structures in the replication enhancer of Flaviviruses and Pestiviruses emerged via convergent evolution. PLoS ONE.

[43] Shurtleff A.C., Beasley D.W.C., Chen J.J.Y., Ni H., Suderman M.T., Wang H., Xu R., Wang E., Weaver S.C., Watts D.M. (2001). Genetic variation in the 3′ non-coding region of dengue viruses. Virology.

[44] Zhou Y., Mammen M.P., Klungthong C., Chinnawirotpisan P., Vaughn D.W., Nimmannitya S., Kalayanarooj S., Holmes E.C., Zhang C. (2006). Comparative analysis reveals no consistent association between the secondary structure of the 3′-untranslated region of dengue viruses and disease syndrome. J. Gen. Virol..

[45] Mandl C.W., Holzmann H., Meixner T., Rauscher S., Stadler P.F., Allison S.L., Heinz F.X. (1998). Spontaneous and Engineered Deletions in the 3′ Noncoding Region of Tick-Borne Encephalitis Virus: Construction of Highly Attenuated Mutants of a Flavivirus. J. Virol..

[46] Leonova G.N., Belikov S.I., Kondratov I.G., Takashima I. (2013). Comprehensive assessment of the genetics and virulence of tick-borne encephalitis virus strains isolated from patients with inapparent and clinical forms of the infection in the Russian Far East. Virology.

[47] Formanová P., Černý J., Bolfíková B.Č., Valdés J.J., Kozlova I., Dzhioev Y., Růžek D. (2015). Full genome sequences and molecular characterization of tick-borne encephalitis virus strains isolated from human patients. Ticks Tick. Borne. Dis..

[48] Gritsun T.S., Gould E.A. (2007). Direct repeats in the flavivirus 3′ untranslated region; a strategy for survival in the environment?. Virology.

[49] Tajima S., Nukui Y., Takasaki T., Kurane I. (2007). Characterization of the variable region in the 3′ non-translated region of dengue type 1 virus. J. Gen. Virol..

[50] Sakai M., Yoshii K., Sunden Y., Yokozawa K., Hirano M., Kariwa H. (2014). Variable region of the 3′ UTR is a critical virulence factor in the Far-Eastern subtype of tick-borne encephalitis virus in a mouse model. J. Gen. Virol..

[51] Pijlman G.P., Funk A., Kondratieva N., Leung J., Torres S., van der Aa L., Liu W.J., Palmenberg A.C., Shi P.Y., Hall R.A. (2008). A Highly Structured, Nuclease-Resistant, Noncoding RNA Produced by Flaviviruses Is Required for Pathogenicity. Cell Host Microbe.

[52] Funk A., Truong K., Nagasaki T., Torres S., Floden N., Balmori Melian E., Edmonds J., Dong H., Shi P.-Y., Khromykh A.A. (2010). RNA Structures Required for Production of Subgenomic Flavivirus RNA. J. Virol..

[53] Silva P.A.G.C., Pereira C.F., Dalebout T.J., Spaan W.J.M., Bredenbeek P.J. (2010). An RNA Pseudoknot Is Required for Production of Yellow Fever Virus Subgenomic RNA by the Host Nuclease XRN1. J. Virol..

[54] Chapman E.G., Moon S.L., Wilusz J., Kieft J.S. (2014). RNA structures that resist degradation by Xrn1 produce a pathogenic dengue virus RNA. Elife.

[55] Moon S.L., Anderson J.R., Kumagai Y., Wilusz C.J., Akira S., Khromykh A.A., Wilusz J. (2012). A noncoding RNA produced by arthropod-borne flaviviruses inhibits the cellular exoribonuclease XRN1 and alters host mRNA stability. RNA.

[56] Chang R.Y., Hsu T.W., Chen Y.L., Liu S.F., Tsai Y.J., Lin Y.T., Chen Y.S., Fan Y.H. (2013). Japanese encephalitis virus non-coding RNA inhibits activation of interferon by blocking nuclear translocation of interferon regulatory factor 3. Vet. Microbiol..

[57] Akiyama B.M., Laurence H.M., Massey A.R., Costantino D.A., Xie X., Yang Y., Shi P.Y., Nix J.C., Beckham J.D., Kieft J.S. (2016). Zika virus produces noncoding RNAs using a multi-pseudoknot structure that confounds a cellular exonuclease. Science.

[58] Chapman E.G., Costantino D.A., Rabe J.L., Moon S.L., Wilusz J., Nix J.C., Kieft J.S. (2014). The structural basis of pathogenic subgenomic flavivirus RNA (sfRNA) production. Science.

[59] Saeedi B.J., Geiss B.J. (2013). Regulation of flavivirus RNA synthesis and capping. Wiley Interdiscip. Rev. RNA.

[60] Dong H., Zhang B., Shi P.Y. (2008). Terminal structures of West Nile virus genomic RNA and their interactions with viral NS5 protein. Virology.

[61] Li X.F., Jiang T., Yu X.D., Deng Y.Q., Zhao H., Zhu Q.Y., De Qin E., Qin C.F. (2010). RNA elements within the 5′ untranslated region of the West Nile virus genome are critical for RNA synthesis and virus replication. J. Gen. Virol..

[62] Zhou Y., Ray D., Zhao Y., Dong H., Ren S., Li Z., Guo Y., Bernard K.A., Shi P.-Y., Li H. (2007). Structure and Function of Flavivirus NS5 Methyltransferase. J. Virol..

[63] Zhang B., Dong H., Zhou Y., Shi P.-Y. (2008). Genetic Interactions among the West Nile Virus Methyltransferase, the RNA-Dependent RNA Polymerase, and the 5′ Stem-Loop of Genomic RNA. J. Virol..

[64] Song Y., Mugavero J., Stauft C.B. (2019). Dengue and Zika Virus 5 = Untranslated Regions Harbor. MBio.

[65] Gebhard L.G., Filomatori C.V., Gamarnik A.V. (2011). Functional RNA elements in the dengue virus genome. Viruses.

[66] Liu Z.Y., Li X.F., Jiang T., Deng Y.Q., Ye Q., Zhao H., Yu J.Y., Qin C.F. (2016). Viral RNA switch mediates the dynamic control of flavivirus replicase recruitment by genome cyclization. Elife.

[67] Sharma S., Varani G. (2020). NMR structure of Dengue West Nile viruses stem-loop B: A key cis-acting element for flavivirus replication. Biochem. Biophys. Res. Commun..

[68] Yakovchuk P., Protozanova E., Frank-Kamenetskii M.D. (2006). Base-stacking and base-pairing contributions into thermal stability of the DNA double helix. Nucleic Acids Res..

[69] Clyde K., Harris E. (2006). RNA secondary structure in the coding region of dengue virus type 2 directs translation start codon selection and is required for viral replication. J. Virol..

[70] Kozak M. (1990). Downstream secondary structure facilitates recognition of initiator codons by eukaryotic ribosomes. Proc. Natl. Acad. Sci. USA.

[71] Clyde K., Barrera J., Harris E. (2008). The capsid-coding region hairpin element (cHP) is a critical determinant of dengue virus and West Nile virus RNA synthesis. Virology.

[72] Mazeaud C., Freppel W., Chatel-Chaix L. (2018). The Multiples Fates of the Flavivirus RNA Genome During Pathogenesis. Front. Genet..

[73] Sanford T.J., Mears H.V., Fajardo T., Locker N., Sweeney T.R. (2019). Circularization of flavivirus genomic RNA inhibits de novo translation initiation. Nucleic Acids Res..

[74] Hahn C.S., Hahn Y.S., Rice C.M., Lee E., Dalgarno L., Strauss E.G., Strauss J.H. (1987). Conserved elements in the 3′ untranslated region of flavivirus RNAs and potential cyclization sequences. J. Mol. Biol..

[75] Men R., Bray M., Clark D., Chanock R.M., Lai C.J. (1996). Dengue type 4 virus mutants containing deletions in the 3′ noncoding region of the RNA genome: Analysis of growth restriction in cell culture and altered viremia pattern and immunogenicity in rhesus monkeys. J. Virol..

[76] Khromykh A.A., Meka H., Guyatt K.J., Westaway E.G. (2001). Essential Role of Cyclization Sequences in Flavivirus RNA Replication. J. Virol..

[77] Alvarez D.E., Lodeiro M.F., Ludueña S.J., Pietrasanta L.I., Gamarnik A.V. (2005). Long-Range RNA-RNA Interactions Circularize the Dengue Virus Genome. J. Virol..

[78] Lo M.K., Tilgner M., Bernard K.A., Shi P.-Y. (2003). Functional Analysis of Mosquito-Borne Flavivirus Conserved Sequence Elements within 3′ Untranslated Region of West Nile Virus by Use of a Reporting Replicon That Differentiates between Viral Translation and RNA Replication. J. Virol..

[79] Zhang B., Dong H., Ye H., Tilgner M., Shi P.Y. (2010). Genetic analysis of West Nile virus containing a complete 3′CSI RNA deletion. Virology.

[80] Olsthoorn R.C.L., Mertens S., Brederode F.T., Bol J.F. (1999). A conformational switch at the 3′ end of a plant virus RNA regulates viral replication. EMBO J..

[81] Pogany J., Fabian M.R., White K.A., Nagy P.D. (2003). A replication silencer element in a plus-strand RNA virus. EMBO J..

[82] Zhang G., Zhang J., Simon A.E. (2004). Repression and Derepression of Minus-Strand Synthesis in a Plus-Strand RNA Virus Replicon. J. Virol..

[83] Zhang G., Zhang J., George A.T., Baumstark T., Simon A.E. (2006). Conformational changes involved in initiation of minus-strand synthesis of a virus-associated RNA. RNA.

[84] Corver J., Lenches E., Smith K., Robison R.A., Sando T., Strauss E.G., Strauss J.H. (2003). Fine Mapping of a cis-Acting Sequence Element in Yellow Fever Virus RNA That Is Required for RNA Replication and Cyclization. J. Virol..

[85] Alvarez D.E., Filomatori C.V., Gamarnik A.V. (2008). Functional analysis of dengue virus cyclization sequences located at the 5′ and 3′UTRs. Virology.

[86] Lott W.B., Doran M.R. (2013). Do RNA viruses require genome cyclisation for replication?. Trends Biochem. Sci..

[87] Welsch S., Miller S., Romero-Brey I., Merz A., Bleck C.K.E., Walther P., Fuller S.D., Antony C., Krijnse-Locker J., Bartenschlager R. (2009). Composition and Three-Dimensional Architecture of the Dengue Virus Replication and Assembly Sites. Cell Host Microbe.

[88] Brinton M.A., Basu M. (2015). Functions of the 3′ and 5′ genome RNA regions of members of the genus Flavivirus. Virus Res..

[89] Rivas E., Clements J., Eddy S.R. (2016). A statistical test for conserved RNA structure shows lack of evidence for structure in lncRNAs. Nat. Methods.

[90] You S., Padmanabhan R. (1999). A novel in vitro replication system for dengue virus: Initiation of RNA synthesis at the 3′-end of exogenous viral RNA templates requires 5′- and 3′- terminal complementary sequence motifs of the viral RNA. J. Biol. Chem..

[91] You S., Falgout B., Markoff L., Padmanabhan R. (2001). In Vitro RNA Synthesis from Exogenous Dengue Viral RNA Templates Requires Long Range Interactions between 5′- and 3′-Terminal Regions that Influence RNA Structure. J. Biol. Chem..

[92] Shi P.Y., Li W., Brinton M.A. (1996). Cell proteins bind specifically to West Nile virus minus-strand 3′ stem-loop RNA. J. Virol..

[93] Blackwell J.L., Brinton M.A. (1997). Translation elongation factor-1 alpha interacts with the 3′ stem-loop region of West Nile virus genomic RNA. J. Virol..

[94] Blackwell J.L., Brinton M.A. (1995). BHK cell proteins that bind to the 3′ stem-loop structure of the West Nile virus genome RNA. J. Virol..

[95] Li D., Wei T., Abbott C.M., Harrich D. (2013). The Unexpected Roles of Eukaryotic Translation Elongation Factors in RNA Virus Replication and Pathogenesis. Microbiol. Mol. Biol. Rev..

[96] Filomatori C.V., Lodeiro M.F., Alvarez D.E., Samsa M.M., Pietrasanta L., Gamarnik A.V. (2006). A 5′ RNA element promotes dengue virus RNA synthesis on a circular genome. Genes Dev..

[97] Lodeiro M.F., Filomatori C.V., Gamarnik A.V. (2008). Structural and Functional Studies of the Promoter Element for Dengue Virus RNA Replication. J. Virol..

[98] Dong H., Zhang B., Shi P.Y. (2008). Flavivirus methyltransferase: A novel antiviral target. Antivir. Res..

[99] Teramoto T., Kohno Y., Mattoo P., Markoff L., Falgout B., Padmanabhan R. (2008). Genome 3′-end repair in dengue virus type 2. RNA.

[100] Selisko B., Potisopon S., Agred R., Priet S., Varlet I., Thillier Y., Sallamand C., Debart F., Vasseur J.J., Canard B. (2012). Molecular Basis for Nucleotide Conservation at the Ends of the Dengue Virus Genome. PLoS Pathog..

[101] Ivanyi-Nagy R., Lavergne J.-P., Gabus C., Ficheux D., Darlix J.-L. (2008). RNA chaperoning and intrinsic disorder in the core proteins of Flaviviridae. Nucleic Acids Res..

[102] Ivanyi-Nagy R., Darlix J.-L. (2012). Core protein-mediated 5′–3′ annealing of the West Nile virus genomic RNA in vitro. Virus Res..

[103] Khromykh A.A., Westaway E.G. (1996). RNA binding properties of core protein of the flavivirus Kunjin. Arch. Virol..

[104] Cristofari G., Darlix J.L. (2002). The ubiquitous nature of RNA chaperone proteins. Prog. Nucleic Acid Res. Mol. Biol..

[105] Pong W.-L., Huang Z.-S., Teoh P.-G., Wang C.-C., Wu H.-N. (2011). RNA binding property and RNA chaperone activity of dengue virus core protein and other viral RNA-interacting proteins. FEBS Lett..

[106] Katoh H., Mori Y., Kambara H., Abe T., Fukuhara T., Morita E., Moriishi K., Kamitani W., Matsuura Y. (2011). Heterogeneous Nuclear Ribonucleoprotein A2 Participates in the Replication of Japanese Encephalitis Virus through an Interaction with Viral Proteins and RNA. J. Virol..

[107] Kaur R., Lal S.K. (2020). The multifarious roles of heterogeneous ribonucleoprotein A1 in viral infections. Rev. Med. Virol..

[108] Gebhard L.G., Kaufman S.B., Gamarnik A.V. (2012). Novel ATP-independent RNA annealing activity of the dengue virus NS3 helicase. PLoS ONE.

[109] Swarbrick C.M.D., Basavannacharya C., Chan K.W.K., Chan S.A., Singh D., Wei N., Phoo W.W., Luo D., Lescar J., Vasudevan S.G. (2017). NS3 helicase from dengue virus specifically recognizes viral RNA sequence to ensure optimal replication. Nucleic Acids Res..

[110] Bidet K., Garcia-Blanco M.A. (2014). Flaviviral RNAs: Weapons and targets in the war between virus and host. Biochem. J..

[111] Vashist S., Anantpadma M., Sharma H., Vrati S. (2009). La protein binds the predicted loop structures in the 3′ non-coding region of Japanese encephalitis virus genome: Role in virus replication. J. Gen. Virol..

[112] Vashist S., Bhullar D., Vrati S. (2011). La protein can simultaneously bind to both 3′- and 5′-noncoding regions of Japanese encephalitis virus genome. DNA Cell Biol..

[113] García-Montalvo B.M., Medina F., Del Angel R.M. (2004). La protein binds to NS5 and NS3 and to the 5′ and 3′ ends of Dengue 4 virus RNA. Virus Res..

[114] Chien H.-L., Liao C.-L., Lin Y.-L. (2011). FUSE Binding Protein 1 Interacts with Untranslated Regions of Japanese Encephalitis Virus RNA and Negatively Regulates Viral Replication. J. Virol..

[115] Lin R.J., Chien H.L., Lin S.Y., Chang B.L., Yu H.P., Tang W.C., Lin Y.L. (2013). MCPIP1 ribonuclease exhibits broad-spectrum antiviral effects through viral RNA binding and degradation. Nucleic Acids Res..

